# Young and lonely? Results from the COMET study

**DOI:** 10.1192/j.eurpsy.2022.1300

**Published:** 2022-09-01

**Authors:** M. Di Vincenzo, L. Marone, A. Del Vecchio, V. Giallonardo, V. Del Vecchio, M. Luciano, G. Sampogna, A. Fiorillo

**Affiliations:** University of Campania “Luigi Vanvitelli”, Department Of Psychiatry, Naples, Italy

**Keywords:** COVID-19 Loneliness Young Lockdown Pandemic Youth, Mental health Covid Mentalhealth Italy

## Abstract

**Introduction:**

The sudden changes in daily routine due to the containment measures adopted for facing the COVID-19 pandemic have had an impact on the mental health of the general population. In particular, young adults are exposed to a higher risk compared to the general population to suffer from the consequences of the pandemic, in terms of anger and irritability, depressive symptoms and somatic complaints, insomnia, lack of motivation and loneliness. In particular, loneliness can be particularly pronounced during young adulthood.

**Objectives:**

This study aimed to describe the levels of loneliness in a sample of Italian young people during the national lockdown in 2020, evaluating clinical and socio-demographic differences and the role of coping strategies and levels of resilience.

**Methods:**

A sub-analysis of a sample of adults aged 18-34 years has been drawn on a larger cross-sectional observational national trial (COMET, 2020) in which, among other psychopathological dimensions, the levels of loneliness have been assessed by the UCLA scale short version.

**Results:**

Levels of loneliness were particularly severe in a third of cases (risk factors: unemployment, low income and vulnerability in mental health), in association with depression, anxiety, stress, OCD symptoms, higher rates of suicidal ideation, sleep disturbance and excessive use of Internet. Levels of loneliness tended to increase over time.

**Conclusions:**

Overall, during the Italian COVID-19 lockdown young people have experienced quite high levels of loneliness: this dimension could represent a useful domain to assess in routine clinical practice.

**Disclosure:**

No significant relationships.

